# Therapeutic hypothermia in patients with acute myocardial infarction complicated by out-of-hospital cardiac arrest

**DOI:** 10.1186/s12916-025-03997-0

**Published:** 2025-03-26

**Authors:** Oh-Hyun Lee, Seok-Jae Heo, Moon-Hyun Kim, Je-Wook Park, SungA Bae, Minkwan Kim, Ji Woong Roh, Yongcheol Kim, Eui Im, In Hyun Jung, Deok-Kyu Cho

**Affiliations:** 1 Division of Cardiology, Department of Internal Medicine, Yonsei University College of Medicine and Cardiovascular Center, Yongin Severance Hospital, Yongin, Republic of Korea; 2 Biostatistics Collaboration Unit, Department of Biomedical Systems Informatics, Yonsei University College of Medicine, Seoul, Republic of Korea

**Keywords:** Out-of-hospital cardiac arrest, Hypothermia, Resuscitation, Prognosism

## Abstract

**Background:**

There is a lack of data regarding outcomes of therapeutic hypothermia in patients with acute myocardial infarction (AMI) complicated by out-of-hospital cardiac arrest (OHCA). This study aimed to evaluate the effect of therapeutic hypothermia on clinical outcomes in comatose patients after percutaneous coronary intervention (PCI) for AMI following OHCA.

**Methods:**

Using a prospective nationwide registry from 2016 to 2021, we selected 2925 patients with AMI who underwent emergency PCI among 182,508 OHCA cases. These patients were divided into groups receiving hypothermia treatment (*n* = 624) and those not receiving hypothermia treatment (*n* = 2301). The primary endpoint was in-hospital mortality, and secondary endpoints were mortality rate at 24 h and neurological outcomes at discharge.

**Results:**

The hypothermia group showed a significantly lower rate of in-hospital mortality than the non-hypothermia group (odds ratio [OR] 0.71; 95% confidence interval [CI], 0.59–0.85; *P* < 0.001). However, there was no significant difference in neurological outcomes at discharge between the two groups. Furthermore, quartile analysis of door-to-cooling (DtC) time, defined as the time from hospital arrival to initiation of hypothermia, demonstrated that a shorter DtC time was associated with a decreased risk of mortality and poor neurological outcomes (mortality: adjusted OR, 0.40; 95% CI, 0.30–0.54; *P* < 0.001; poor neurological outcome: adjusted OR, 0.59; 95% CI, 0.45–0.77; *P* < 0.001 for quartile 1 versus quartile 4).

**Conclusions:**

Therapeutic hypothermia reduced the rate of in-hospital mortality in patients with AMI complicated by OHCA. Moreover, early initiation of hypothermia demonstrated a reduction in mortality and poor neurological outcomes.

**Pre-registered clinical trial number:**

URL: http://clinicaltrials.gov. Unique identifier: NCT05724914.

**Condensed abstract:**

In this large, government-controlled, nationwide, prospective real-world registry with AMI and complicated by OHCA, we demonstrated therapeutic hypothermia reduced the rate of in-hospital mortality, but it did not improve neurological outcomes at discharge. Our findings also showed that early initiation of hypothermia was significantly associated with reduced in-hospital mortality and poor neurological outcomes.

The findings of this study suggest that therapeutic hypothermia reduces in-hospital mortality in patients with AMI complicated by OHCA*. *Early application of hypothermia should be considered as a potential means of improving neurological outcomes in patients with AMI-OHCA undergoing emergency PCI.

**Graphical Abstract:**

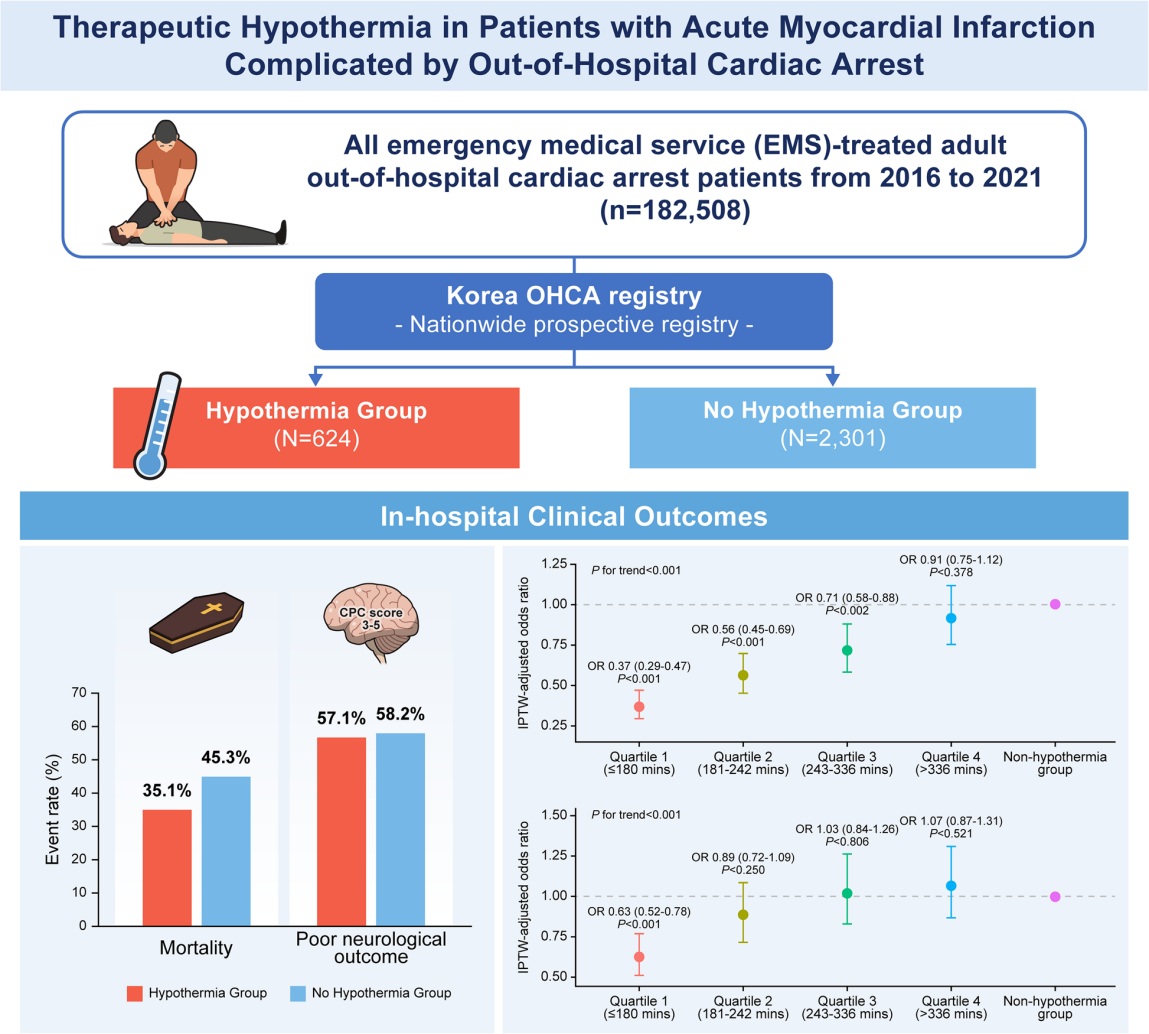

**Supplementary Information:**

The online version contains supplementary material available at 10.1186/s12916-025-03997-0.

## Background

The majority of adult cardiac arrests are associated with obstructive coronary artery disease [1]. Therefore, current guidelines recommend emergency revascularization in all patients with resuscitated cardiac arrest and ST-segment elevation on electrocardiography, as well as in patients with resuscitated cardiac arrest without ST-segment elevation but with a high probability of acute coronary occlusion [2–4].

Acute myocardial infarction (AMI) is associated with high mortality and remains a significant public health issue. Despite advances in reperfusion therapy, the mortality rate for patients with ST-segment elevation myocardial infarction and cardiogenic shock is around 50% [5]. Additionally, outcomes for these patients have not improved in the past two decades [6, 7]. AMI complicated by out-of-hospital cardiac arrest (AMI-OHCA) is particularly serious, with equal mortality to patients in cardiogenic shock and ten times the mortality of patients without cardiac arrest complications [8, 9].

Regardless of the clinical presentation of OHCA, a significant number of AMI patients still experience extensive necrosis even after prompt blood flow restoration. In animal studies, hypothermia has been used in AMI patients to reduce cardiac energy consumption, which has been associated with reduced infarct size [10]. However, randomized control trials and meta-analyses of therapeutic hypothermia after percutaneous coronary intervention (PCI) have not consistently shown significant benefits compared to standard care without hypothermia [11–18].

There is limited data on the clinical impact of therapeutic hypothermia in AMI patients with complications of OHCA. Therefore, this study aimed to evaluate the effect of therapeutic hypothermia on clinical outcomes in comatose patients after PCI for AMI following OHCA.

## Methods

### Study protocols

The data for this study were collected from the Korea OHCA Registry, which includes all patients with cardiac arrest transported by emergency medical services (EMS). The registry was established by the Korea Disease Control and Prevention Agency (KDCA) in collaboration with the National Fire Agency in 2008. It is a nationwide, prospective database that includes all cases transported through EMS to approximately 600 hospitals in the Republic of Korea, following the updated Utstein style (Additional file 1: Table S1) [19]. The National Fire Agency identifies OHCA patients and provides prehospital information by integrating various EMS records, while the KDCA collects hospital information and clinical outcomes through a medical record review. Previous reports have provided detailed information on quality management protocols, the comprehensive data collection process, and explanations of the registry [20]. This study was approved by the institutional review boards of the Yonsei University Yongin Severance Hospital (9–2022-0042) and the Korea Centers for Disease Control and Prevention (KDCA-12–02-CA-2023–000001), and the results of the study are independent and not directly affiliated with the Centers for Disease Control and Prevention.

### Study population and definition

**Fig. 1:**
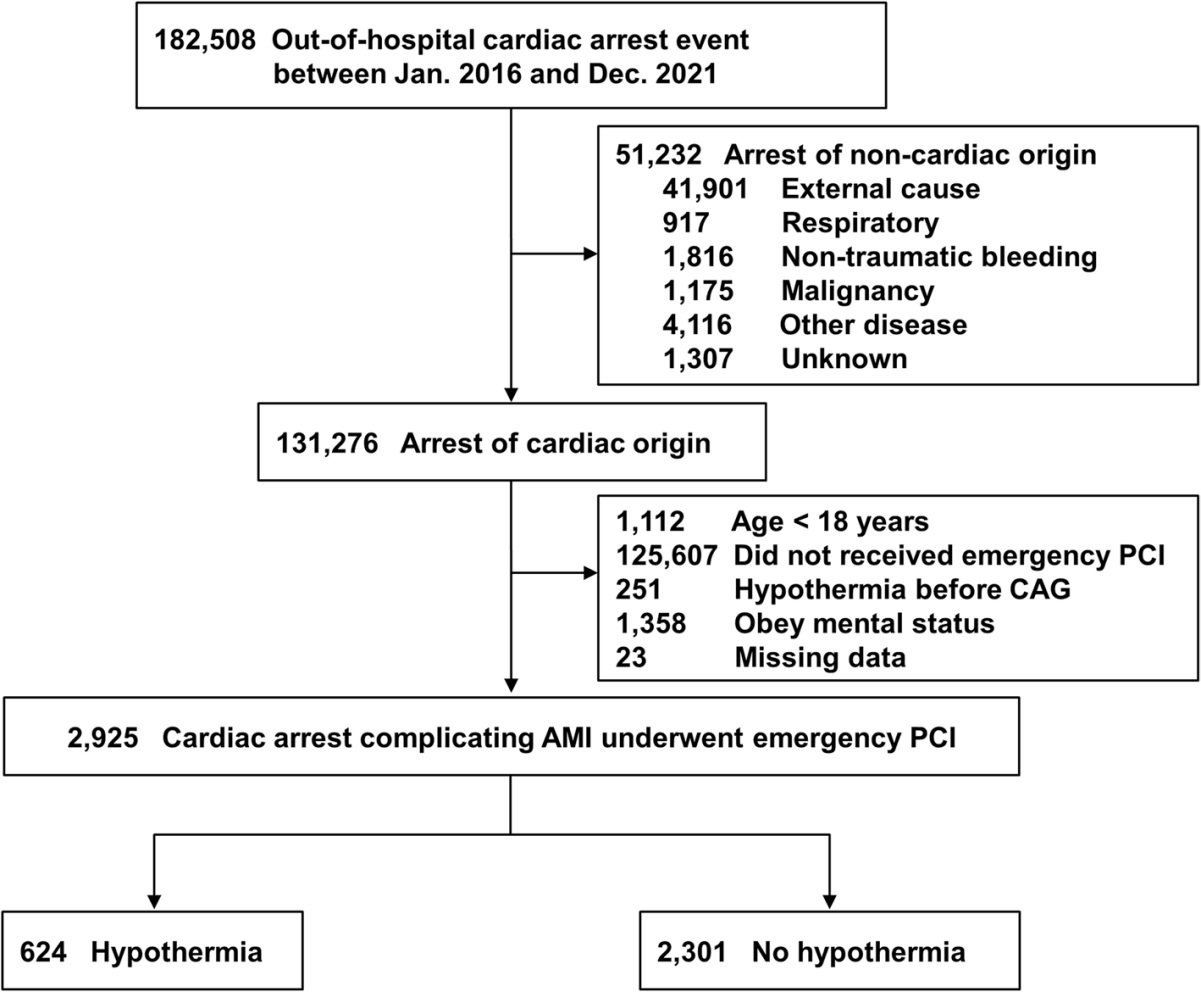
Study flowchart. AMI, acute myocardial infarction; CAG, coronary angiography; CPR, cardiopulmonary resuscitation; CtD, call-to-door time; PCI, percutaneous coronary intervention

Among 182,508 consecutive OHCA patients, we selected 131,276 patients with cardiac arrest enrolled between January 2016 and December 2021 (Fig. 1). The exclusion criteria for this study were patients with non-cardiac arrest causes (external causes including asphyxia, hanging, falls, drowning, road traffic injuries, drug overdose, respiratory issues, non-traumatic bleeding, malignancy, stroke, other diseases, and unknown causes) (*n* = 51,232); patients younger than 18 years (*n* = 1112); patients who did not receive emergency PCI (*n* = 125,607); patients treated with therapeutic hypothermia prior to PCI (*n* = 251); patients without severe brain injury defined as those with a Glasgow Coma Scale [21] of 9 or greater after return of spontaneous circulation (ROSC) (*n* = 1358); and cases with missing data for hypothermia or PCI (*n* = 23). A total of 2925 subjects were divided into two groups based on the administration of therapeutic hypothermia: the hypothermia group (*n* = 624) and the non-hypothermia group (*n* = 2301).

### Clinical outcomes

The primary outcome of the study was death from any cause at the time of hospital discharge. Secondary outcomes included mortality rates at 24 h and poor neurological outcomes, which were defined using Glasgow-Pittsburgh Cerebral Performance Category (CPC) scores at the time of hospital discharge. The CPC score categorized neurological outcomes as follows: CPC1: full recovery; CPC2: moderate disability; CPC3: severe disability; CPC4: coma or vegetative state; and CPC5: death. A good neurological outcome was defined as a CPC score of 1 or 2, while a poor neurological outcome was defined as a CPC score of 3 to 5 [19]. Additionally, the study analyzed the time from hospital arrival to initiation of hypothermia (door-to-cooling time, DtC) in quartiles, as well as the mortality and neurological outcomes of patients treated with hypothermia across quartiles. Furthermore, the study compared specific therapeutic hypothermia protocols, including a target temperature of 33 °C versus 36 °C and a duration of 48 h versus 24 h.

### Statistical analysis

All data were presented as mean ± standard deviation (SD) or median (interquartile range) for continuous variables and frequency (percentage) for categorical variables. The normality assumption of the continuous variables was assessed using the Shapiro–Wilk test and graphical method, such as histogram and quantile–quantile plot. The independent two-sample *t*-test was used to compare differences in continuous variables between the two groups. The chi-squared or Fisher's exact test was used to compare differences in categorical variables between the two groups based on the number of events. Survival probability was estimated using the Kaplan–Meier method, and survival curves were compared between the hypothermia and non-hypothermia groups using the log-rank test. Logistic regression models (adjusted for covariates) were used to assess clinical outcomes. Variables included in the multivariable analysis were selected if they showed significant differences between the two groups (*P* < 0.05) or had predictive value (e.g., age, sex). Inverse probability of treatment weighting (IPTW) was also used to account for confounding factors. The propensity score (PS) was estimated using multiple logistic regression analysis with all covariates. The standardized mean difference (SMD) was used to assess the balance of covariate distribution between the groups, with an SMD < 0.1 considered balanced. Data manipulation and statistical analyses were performed using SAS version 9.3 (SAS Institute) and R software (version 4.1.1; R Foundation for Statistical Computing, Vienna, Austria). Statistical significance was set at *P* < 0.05.

## Results

### Baseline characteristics

The final analysis included 2925 patients with OHCA. Their mean age was 62.1 ± 12.2 years, and 86.6% of them were male. Out of these patients, 624 (21.3%) received therapeutic hypothermia, while the remaining 2301 (78.7%) did not. The hypothermia group was younger and had a higher proportion of male patients than did the non-hypothermia group. In the hypothermia group, the median DtC time was 241.5 min (interquartile range, 62–2046 min).

The prevalence of ischemic heart disease was lower in the hypothermia group than in the non-hypothermia group. The baseline clinical, cardiac arrest, and therapeutic characteristics of the two groups are summarized in Table 1. Healthcare professionals were less common as the first responders in the hypothermia group. Shockable rhythm as the initial EMS rhythm was detected in 79.3% of patients in the hypothermia group and 72.1% of patients in the non-hypothermia group. The use of automated external defibrillators was higher in the hypothermia group. ROSC was achieved in 45.0% of patients in the hypothermia group and 45.2% in the non-hypothermia group. After IPTW adjustment, the SMD between the groups was < 0.01 for all variables, indicating adequate adjustment. There were no significant differences in the baseline characteristics between the groups in the IPTW-adjusted population (Additional file 1: Table S2 and Fig. S1).

**Table 1:** Baseline characteristics

	Total (*N* = 2925)	Hypothermia (*N* = 624)	No hypothermia (*N* = 2301)	*P* value
Demographics				
Age, years	62.1 ± 12.2	60.7 ± 11.9	62.6 ± 12.3	< 0.001
< 50	442 (15.1)	109 (17.5)	333 (14.5)	
50 ~ 70	1673 (57.2)	369 (59.1)	1304 (56.7)	
≥ 70	810 (27.7)	146 (23.4)	664 (28.9)	
Male	2533 (86.6)	560 (89.7)	1973 (85.7)	0.011
Comorbidities				
Hypertension	1321 (45.2)	273 (43.8)	1048 (45.5)	0.451
Diabetes mellitus	849 (29.0)	165 (26.4)	684 (29.7)	0.120
Cardiovascular disease	659 (22.5)	120 (19.2)	539 (23.4)	0.030
Ischemic heart disease	561 (19.2)	100 (16.0)	461 (20.0)	0.028
Valvular heart disease	13 (0.4)	1 (0.2)	12 (0.5)	0.388
Arrhythmia	95 (3.2)	17 (2.7)	78 (3.4)	0.481
Heart failure	66 (2.3)	13 (2.1)	53 (2.3)	0.860
Cerebrovascular disease	227 (7.8)	40 (6.4)	187 (8.1)	0.181
Chronic kidney disease	164 (5.6)	26 (4.2)	138 (6.0)	0.096
Chronic lung disease	91 (3.1)	19 (3.0)	72 (3.1)	1.000
Characteristics of the cardiac arrest				
Place at cardiac arrest				0.971
Public place	996 (34.1)	215 (34.5)	781 (33.9)	
Non-public place	1312 (44.9)	278 (44.6)	1034 (44.9)	
Unknown	617 (21.1)	131 (21.0)	486 (21.1)	
Witness type				0.009
Healthcare professional	478 (16.3)	79 (12.7)	399 (17.3)	
Bystander	1745 (59.7)	400 (64.1)	1345 (58.5)	
Unknown	702 (24.0)	145 (23.2)	557 (24.2)	
Layperson witnessed	2298 (78.6)	1800 (78.2)	498 (79.8)	0.425
Bystander-performed CPR	1505 (51.5)	1172 (50.9)	333 (53.4)	0.302
Telemetric advice before EMS	2001 (68.4)	1565 (68.0)	436 (69.9)	0.403
Initial EMS rhythm				0.001
Shockable rhythm	2155 (73.7)	495 (79.3)	1660 (72.1)	
Nonshockable rhythm	726 (24.8)	123 (19.7)	603 (26.2)	
Unknown	44 (1.5)	6 (1.0)	38 (1.7)	
AED use	2270 (77.6)	520 (83.3)	1750 (76.1)	< 0.001
Arres to admission timeline				
Arrest-to-call time, min	0 (0–2)	0 (0–2)	0 (0–2)	0.235
Call-to-door time, min	30 (24–38)	31 (26–38)	30 (24–38)	0.005
Arrest-to-door time, min	30 (24–38)	31 (26–38)	30 (24–38)	0.005
PCI timeline				
Door-to-PCI time, min	100 (74–146)	100 (74–140)	100 (74–148)	0.492
Procedure time, min	25 (15–41)	23 (14–40)	25 (15–41)	0.275
Characteristics on ER admission				
Initial ECG rhythm				0.344
Post ROSC rhythm	1450 (49.6)	310 (49.7)	1140 (49.5)	
Shockable rhythm	335 (11.5)	81 (13.0)	254 (11.0)	
Nonshockable rhythm	859 (29.4)	182 (29.2)	677 (29.4)	
No record	281 (9.6)	51 (8.2)	230 (10.0)	
Defibrillation at ER	968 (33.1)	213 (34.1)	755 (32.8)	0.565
Total CPR time at ER, min	4 (0–19)	4 (0–18)	4 (0–19)	0.441
ROSC before ER admission	1321 (45.2)	281 (45.0)	1040 (45.2)	0.977
Therapeutic interventions				
ECMO	612 (20.9)	136 (21.8)	476 (20.7)	0.584

Values are mean ± SD, *n* (%) or median (interquartile range)

*AED* Automated external defibrillator, *CPR* Cardiopulmonary resuscitation, *ECG* Electrocardiogram, *ECMO* Extracorporeal membrane oxygenation, *EMS* Emergency medical service, *ER* Emergency room, *PCI* Percutaneous coronary intervention, *ROSC* Return of spontaneous circulation

### In-hospital mortality and neurological outcomes

**Fig. 2:**
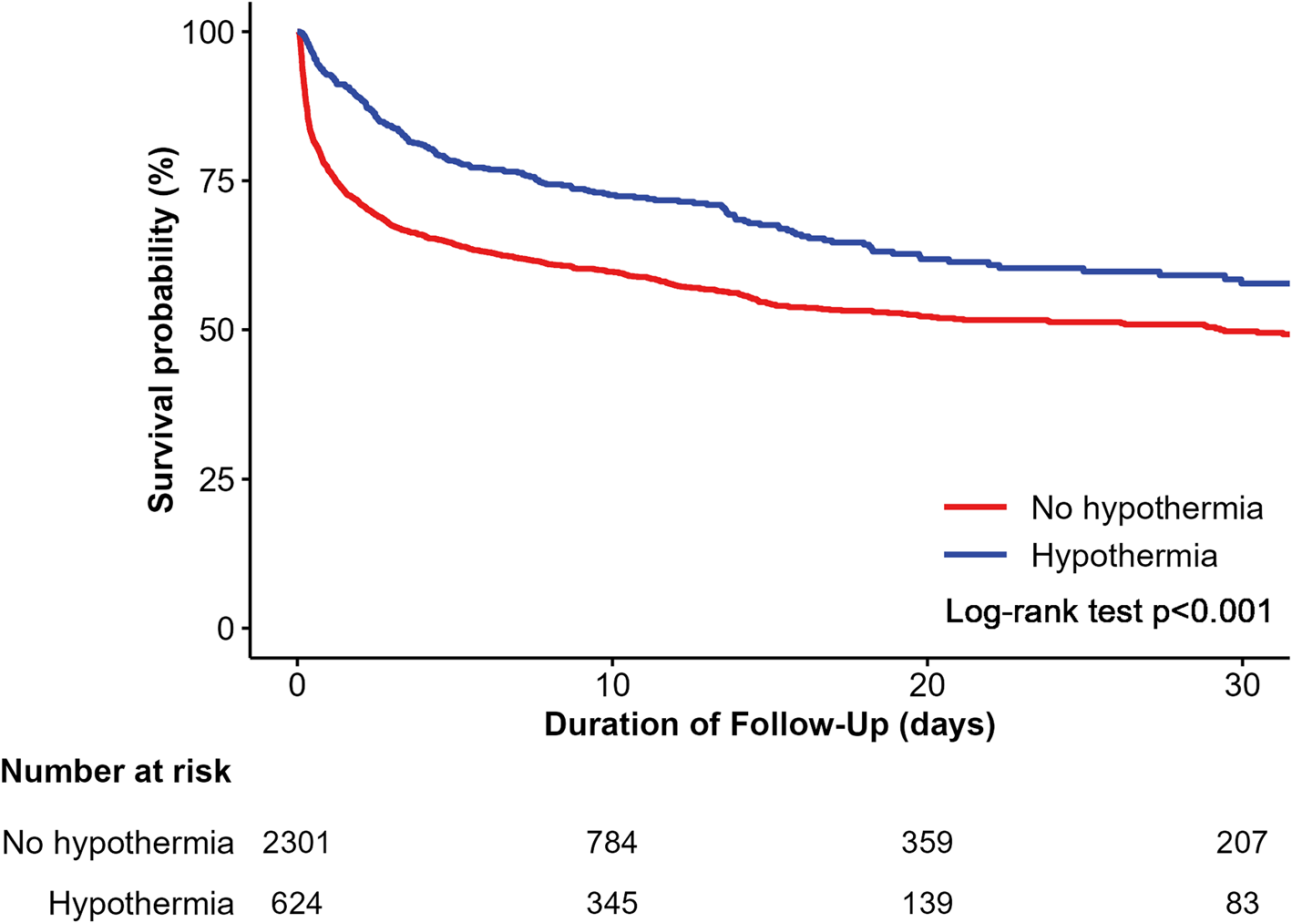
Kaplan–Meier curves comparing therapeutic-hypothermia and non-hypothermia group survival

The primary outcome of all-cause death at hospital discharge was observed in 219 (35.1%) and 997 (43.3%) patients in the hypothermia and non-hypothermia groups, respectively (odds ratio (OR), 0.71; 95% confidence interval [CI], 0.59–0.85; *P* < 0.001) (Table 2 and Fig. 2). Mortality rates within 24 h were also significantly lower in the hypothermia group than in the non-hypothermia group (OR, 0.26; 95% CI, 0.19–0.35; *P* < 0.001). However, there were no significant differences in neurological outcomes at discharge in either group. These results were consistent after multiple sensitivity analyses using multivariable Cox regression analyses and performing IPTW adjustments. These findings were consistent across most subgroups, with lower in-hospital mortality rates in the hypothermia group, while neurological outcomes showed no significant difference between the two groups (Additional file 1: Fig. S2).

**Table 2:** Comparison of in-hospital clinical outcomes

	Hypothermia (*N*=624)	No hypothermia (*N*=2301)	Unadjusted		Multivariable-adjusted^*^		IPTW-adjusted	
			OR (95% CI)	*P* value	HR (95% CI)	*P* value	HR (95% CI)	*P* value
Mortality outcomes								
Death within discharge	219 (35.1)	997 (43.3)	0.71 (0.59–0.85)	<0.001	0.72 (0.57–0.91)	0.005	0.78 (0.70–0.87)	<0.001
Death within 24 h	45 (7.2)	533 (23.2)	0.26 (0.19–0.35)	<0.001	0.24 (0.17–0.34)	<0.001	0.33 (0.28–0.38)	<0.001
Neurologic outcomes								
Poor outcome (CPC 3, 4, 5)	356 (57.1)	1,339 (58.2)	0.95 (0.80–1.14)	0.609	1.06 (0.84–1.33)	0.637	1.01 (0.91–1.13)	0.824

^*^Adjusted variable: age, sex, location at cardiac arrest, bystander-witnessed, bystander-performed CPR, telemetric advice to first aid before EMS, initial EMS rhythm, defibrillation before ER admission, ROSC before ER admission, initial ECG rhythm, defibrillation, total CPR time, defibrillation at ER, ECMO, and hospital region

Values are *n* (%, cumulative incidence) unless otherwise indicated

### Clinical outcomes according to DtC time in patients receiving therapeutic hypothermia

**Fig. 3:**
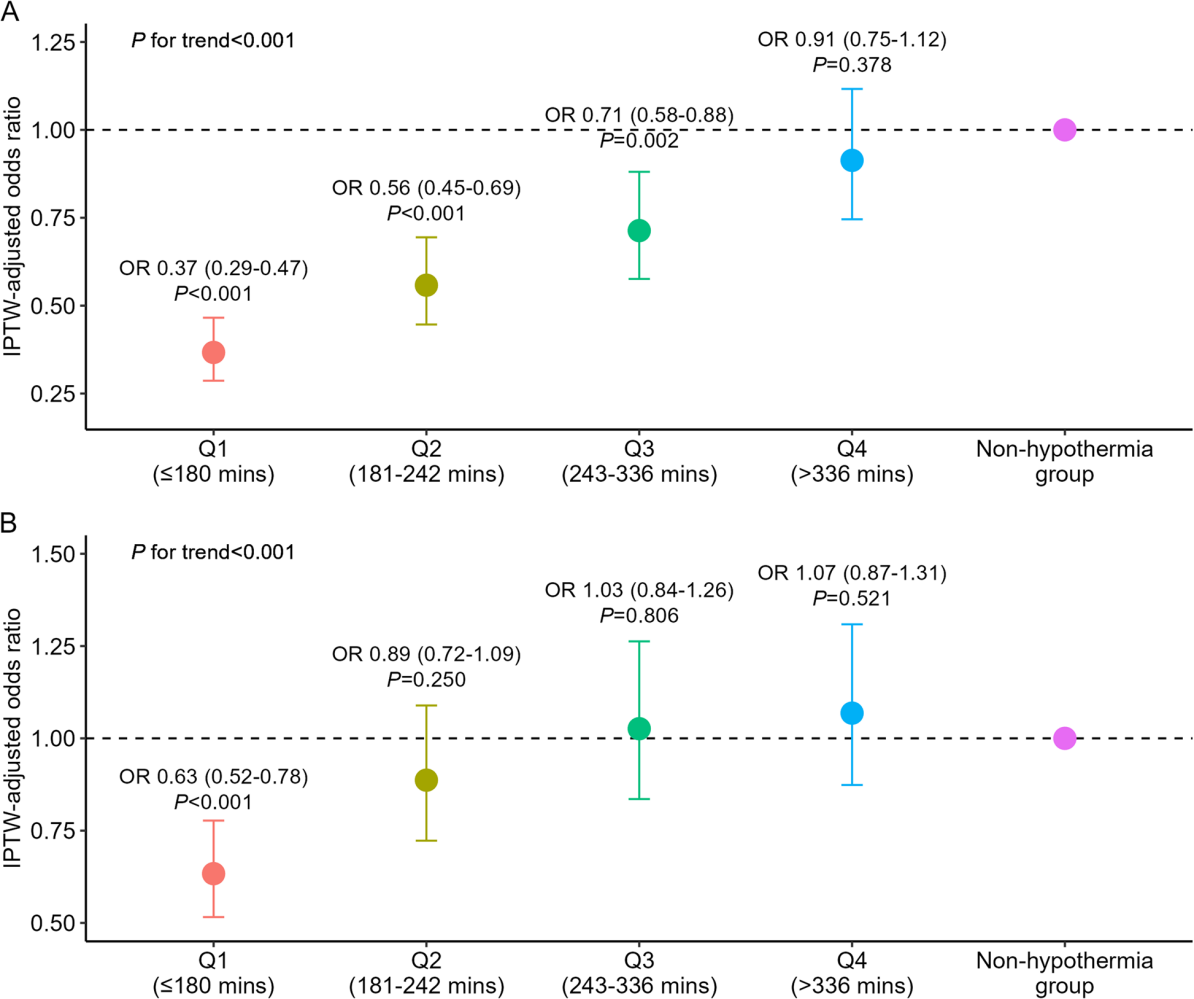
Comparison of mortality (**A**) and poor neurologic outcomes (**B**) based on hypothermia initiation time. IPTW, inverse probability of treatment weighting; OR, odds ratio

In patients who received therapeutic hypothermia, the DtC times were divided into quartiles to compare clinical outcomes, including in-hospital mortality and neurological outcomes, with those of the non-hypothermia group (Q1, ≤ 180 min; Q2, 181–242 min; Q3, 243–336 min; Q4, > 336 min). The odds ratios of the DtC time quartiles for clinical outcomes are shown in Fig. 3. Quartile 1 was significantly associated with a reduction in in-hospital mortality and poor neurological outcomes than quartile 4 (mortality: adjusted OR, 0.40; 95% CI, 0.30–0.54; *P* < 0.001; poor neurological outcome: adjusted OR, 0.59; 95% CI, 0.45–0.77; *P* < 0.001 for quartile 1 versus quartile 4). In addition, shorter DtC times were associated with a decreased risk of in-hospital mortality and poor neurological outcomes (*P* < 0.001 for the trend across quartiles for both mortality and poor neurological outcomes).

### Comparison of hypothermia protocol in patients receiving therapeutic hypothermia

Among the 624 patients who received hypothermia treatment, 509 successfully maintained the target temperature for at least 24 h. Of a total of 624 patients who received therapeutic hypothermia, 523 (83.8%) and 101 (16.2%) patients received hypothermia therapy at 33 °C and 36 °C, respectively. There were no significant differences in in-hospital mortality (OR, 0.97; 95% CI, 0.63–1.53; *P* = 0.900) and poor neurological outcomes (OR, 1.13; 95% CI, 0.74–1.74; *P* = 0.565) between the two groups before and after multiple sensitivity analyses (Additional file 1: Table S3).

Regarding the clinical outcomes based on the duration of therapeutic hypothermia, 125 patients had missing data on the duration of hypothermia. Among the remaining 499 patients, 374 (74.9%) received therapeutic hypothermia for 24 h and 125 (25.1%) received therapeutic hypothermia for 48 h. The risks of in-hospital mortality and poor neurological outcomes were comparable between the groups in both patients who received therapeutic hypothermia for 24 h (OR, 1.06; 95% CI, 0.67–1.66; *P* = 0.786) and 48 h (OR, 0.91; 95% CI, 0.61–1.37; *P* = 0.660) before and after multiple sensitivity analyses (Additional file 1: Table S4).

## Discussion

In this study, we investigated the differences in in-hospital clinical outcomes based on the application of hypothermia in patients with AMI-OHCA. The main findings can be summarized as follows: First, therapeutic hypothermia was associated with a significant reduction in in-hospital mortality in patients with AMI-OHCA. However, it did not reduce the incidence of poor neurological outcomes. Second, quartile analysis of DtC time demonstrated that shorter DtC times were associated with a significantly decreased risk of in-hospital mortality and poor neurological outcomes in the therapeutic hypothermia group. Third, a detailed hypothermia protocol with a target temperature of 33 °C vs. 36 °C or a hypothermia duration of 24 h vs. 48 h did not show a significant difference in the rate of in-hospital mortality and poor neurological outcomes at discharge.

To date, several randomized trials regarding hypothermia have been conducted with heterogeneous study populations and designs, yielding inconclusive results (Additional file 1: Table S5) [22–27]. With regard to the study population, research on therapeutic hypothermia has focused primarily on two discrete groups: all patients who have experienced OHCA or patients with AMI who have neither experienced OHCA nor are in cardiogenic shock. Only a few studies have been published on hypothermia in patients with AMI and cardiogenic shock (CS): a study in which hypothermia did not increase the incidence of stent thrombosis after PCI in patients with AMI and CS from the US national database [28], and a randomized trial of the effect of hypothermia on cardiac power index values at 24 h after primary PCI in patients with AMI presenting as CS [16]. Nevertheless, there is a notable lack of studies evaluating mortality and neurological outcomes after therapeutic hypothermia specifically in patients with AMI-OHCA. Therefore, our study aimed to assess the impact of therapeutic hypothermia on mortality and neurologic outcomes in patients with AMI-OHCA using large-scale, prospective real-world registry data.

Two randomized trials on the impact of hypothermia on OHCA patients with an initial shockable rhythm were published in 2002 [22, 23]. The first study involved 43 patients who underwent hypothermia at 33℃ within 2 h of ROSC and showed neurological benefits compared to the 34 patients treated with normothermia (37℃). The second study, the HACA (Hypothermia after Cardiac Arrest Study) trial, demonstrated mortality benefits in 137 patients who underwent hypothermia at 32–34℃ compared to the 138 patients treated with normothermia. More recently, the HYPERION (Therapeutic Hypothermia after Cardiac Arrest in Non-shockable Rhythm) trial, which involved 584 patients with cardiac arrest and a non-shockable rhythm, showed better neurological outcomes with targeted hypothermia at 33℃ than with targeted normothermia at 37℃ [26]. As a result, current guidelines recommend therapeutic hypothermia within a target range of 32℃ to 36℃ for comatose adults after ROSC from OHCA, regardless of the initial rhythm, although the evidence supporting this recommendation is not strong [3]. However, a subsequent randomized TTM2 (Targeted Hypothermia versus Targeted Normothermia after Out-of-Hospital Cardiac Arrest) trial showed that therapeutic hypothermia at 33℃ did not lead to a lower incidence of death by 6 months compared to targeted normothermia at 37.5℃ in 1900 patients with coma after OHCA [27]. Despite these conflicting results, our study demonstrated that the hypothermia group had a significantly lower rate of in-hospital mortality than did the non-hypothermia group among patients with AMI-OHCA. Therefore, even with previous studies presenting mixed results, it is essential for physicians to recognize that hypothermia is associated with reduced mortality in OHCA survivors, especially in patients with OHCA-AMI who undergo emergency PCI.

Neurologic impairment is a significant complication to consider for patients who are successfully resuscitated after cardiac arrest. In this context, pre-clinical data provides robust evidence of the neuroprotective effect of hypothermia. Specifically, previous studies have shown that hypothermia reduces cerebral metabolic oxygen demand [29] and decreases ischemia-induced apoptosis. However, the results of clinical trials have been inconsistent. The HACA trial demonstrated that hypothermia increased the rate of favorable neurologic outcomes in patients with cardiac arrest due to ventricular fibrillation [23]. Belliard et al. also found that hypothermia significantly improved neurologic outcomes in patients with OHCA and shockable rhythm [30]. On the other hand, the TTM (targeted temperature management at 33℃ versus 36℃ after cardiac arrest) trial failed to show the neuroprotective effect of hypothermia in patients with OHCA [24], and the HYPERION trial also failed to demonstrate a favorable neurological outcome of hypothermia in patients with OHCA and non-shockable rhythms [26]. Our study, aligning with these trials, showed that therapeutic hypothermia did not result in a more favorable neurological outcome compared to the non-hypothermia group. However, when conducting a quartile analysis of DtC time, we found that early application of hypothermia after hospital arrival was associated with a lower incidence of poor neurological outcomes. Nevertheless, the mechanism behind this phenomenon is poorly understood and warrants large-scale randomized trials. Overall, based on the results of our study, we suggest that hypothermia should be applied promptly to improve neurological outcomes in patients with AMI-OHCA undergoing emergency PCI.

Regarding a detailed hypothermia protocol, the TTM trial showed that more intensive hypothermia at 33 ℃ did not result in improved mortality or neurological outcomes when compared to the use of 36 ℃ [24]. Similarly, in the HYPERION and TTM2 trials, the application of hypothermia at the lower target temperature of 33℃ did not demonstrate a favorable impact on mortality or neurological outcomes when compared to the hypothermia at 37℃ and 37.5℃, respectively [26, 27]. Consistent with these findings, the current study showed that a target temperature of 33℃ did not affect prognosis when compared with that at 36℃. Furthermore, in terms of the duration of hypothermia, the TTH48 (targeted temperature management for 48 vs 24 h and neurological outcome after out-of-hospital cardiac arrest) trial showed that hypothermia at 33℃ for 48 h did not significantly improve 6-month neurological outcomes compared to hypothermia at 33℃ for 24 h [25].

Owing to lack of sufficient evidence, current guidelines recommend maintaining therapeutic hypothermia for at least 24 h after the target temperature is achieved, a recommendation classified as Class 2A. The current study also showed that the duration of hypothermia did not improve mortality and neurological outcomes. In response to these gaps, the ongoing Influence of Cooling Duration on Efficacy in Cardiac Arrest Patients (ICECAP) trial (NCT04217551) is currently enrolling up to 1800 adult comatose survivors after OHCA to determine the optimal duration (e.g., 12, 24, or 48 h) of temperature management to achieve good neurological outcomes. [31] Thus, further research is required to identify the optimal protocol for therapeutic hypothermia following OHCA in order to improve outcomes.

### Study limitations

Our study has several limitations. First, its non-randomized, observational design has inherent selection and information biases. Furthermore, there was a large disparity in the number of patients between the two groups. However, logistic regression analyses with multivariable and IPTW were performed to adjust for measured and unmeasured confounders. Second, we did not consider the diversity of therapeutic hypothermia protocols by institution when analyzing the results. To address this concern, we performed an additional analysis by adjusting for these regional differences of hospital and reassessed the main outcome accordingly. Third, detailed information on AMI including CAD extent and PCI procedures, including the use of intravascular imaging modalities and post-procedure medication use, was not assessed. Additionally, laboratory findings such as CK-MB, cardiac troponin, lactate, or pH levels were not provided. Fourth, beyond target temperature and hypothermia duration, we did not analyze the specific techniques or protocols utilized for hypothermia treatment. Fifth, changes in consciousness levels during the treatment period were not assessed, which could be a potential confounder in our outcome analysis. Sixth, the economic impact of hypothermia treatment could not be evaluated in this study, as our dataset did not include the necessary cost-related variables for such analysis. Finally, we did not evaluate long-term clinical and neurological outcomes.

## Conclusions

In patients with AMI-OHCA who underwent emergency PCI, therapeutic hypothermia was associated with a significant reduction in the rate of in-hospital mortality, but not in neurologic outcomes at discharge. Notably, early initiation of hypothermia demonstrated a significantly decreased risk of mortality and poor neurological outcomes in hospitals in patients receiving therapeutic hypothermia.

## Supplementary Information

Additional file 1: Table S1-S5 and Figure S1-S2. Table S1. Annual trends of ambulance transfers and medical record investigations for patients with out-of-hospital cardiac arrest in Republic of Korea. Table S2. Baseline characteristics after inverse probability of treatment weighting. Table S3. Comparison of clinical outcome according to the target temperature. Table S4. Comparison of clinical outcome according to the duration of therapeutic hypothermia. Table S5. Key randomized controlled trials on hypothermia in patients with OHCA. Figure S1. Propensity score distribution before (A) and after IPTW (B). Figure S2. Subgroup analysis for in-hospital mortality and poor neurological outcomes

## Data Availability

No datasets were generated or analysed during the current study.

## Abbreviations

AMI: Acute myocardial infarction
OHCA: Out-of-hospital cardiac arrest
PCI: Percutaneous coronary intervention
EMS: Emergency medical services
CPC: Cerebral performance category
DtC: Door-to-cooling time
