# Supplementary material for: Therapeutic hypothermia in patients with acute myocardial infarction complicated by out-of-hospital cardiac arrest
Source: BMC Med. 2025 Mar 26;23:179. doi: 10.1186/s12916-025-03997-0 (PMC11948712; doi:10.1186/s12916-025-03997-0)
Supplement: Supplementary file 1 — Additional file 1: Table S1-S5 and Figure S1-S2. Table S1. Annual trends of ambulance transfers and medical record investigations for patients with out-of-hospital cardiac arrest in Republic of Korea. Table S2. Baseline characteristics after inverse probability of treatment weighting. Table S3. Comparison of clinical outcome according to the target temperature. Table S4. Comparison of clinical outcome according to the duration of therapeutic hypothermia. Table S5. Key randomized controlled trials on hypothermia in patients with OHCA. Figure S1. Propensity score distribution before (A) and after IPTW (B). Figure S2. Subgroup analysis for in-hospital mortality and poor neurological outcomes [file 12916_2025_3997_MOESM1_ESM.docx]

**Additional file 1**

**Therapeutic Hypothermia in Patients with Acute Myocardial Infarction Complicated by Out-of-Hospital Cardiac Arrest**

Oh-Hyun Lee, MD; Seok-Jae Heo, PhD; Moon-Hyun Kim, MD; Je-Wook Park, MD; SungA Bae, MD; Minkwan Kim, MD; Ji Woong Roh, MD; Yongcheol Kim, MD; Eui Im, MD; In Hyun Jung, MD; Deok-Kyu Cho, MD

**CONTENTS**

**Table S1. Annual trends of ambulance transfers and medical record investigations for patients with out-of-hospital cardiac arrest in Republic of Korea**.......................................1

**Table S2. Baseline characteristics after inverse probability of treatment weighting**.........2

**Table S3. Comparison of clinical outcome according to the target temperature**……...…5

**Table S4. Comparison of clinical outcome according to the duration of therapeutic hypothermia**…………………………………………………………………….…………….6

**Table S5.** **Key randomized controlled trials on hypothermia in patients with OHCA**…7

**Figure S1. Propensity score distribution before (A) and after IPTW (B)**.........................9

**Figure S2. Subgroup analysis for in-hospital mortality and poor neurological outcomes**.................................................................................................................................10

**Table S1. Annual trends of ambulance transfers and medical record investigations for patients with out-of-hospital cardiac arrest in Republic of Korea**

|  | | 2006 | 2007 | 2008 | 2009 | 2010 | 2011 | 2012 | 2013 |
| --- | --- | --- | --- | --- | --- | --- | --- | --- | --- |
| Ambulance Transfer | hospitals | 812 | 757 | 708 | 742 | 757 | 739 | 712 | 661 |
|  | patients | 19,480 | 20,353 | 21,905 | 24,442 | 25,909 | 26,382 | 27,823 | 29,356 |
| Medical record investigation | hospitals | 616 | 619 | 634 | 623 | 644 | 585 | 593 | 575 |
|  | patients | 16,348 | 18,060 | 20,091 | 22,667 | 24,479 | 24,902 | 26,531 | 28,170 |

|  | | 2014 | 2015 | 2016 | 2017 | 2018 | 2019 | 2020 | 2021 |
| --- | --- | --- | --- | --- | --- | --- | --- | --- | --- |
| Ambulance Transfer | hospitals | 644 | 590 | 556 | 536 | 492 | 476 | 454 | 440 |
|  | patients | 30,309 | 30,771 | 29,832 | 29,262 | 30,539 | 30,782 | 31,652 | 33,235 |
| Medical record investigation | hospitals | 566 | 547 | 505 | 514 | 477 | 458 | 446 | 433 |
|  | patients | 29,282 | 29,959 | 28,963 | 28,629 | 30,179 | 30,279 | 31,417 | 33,041 |

**Table S2. Baseline characteristics after inverse probability of treatment weighting**

|  | **Hypothermia (N=624)** | **No hypothermia (N=2,301)** | **SMD** |
| --- | --- | --- | --- |
| Demographics |  |  |  |
| Age, y | 62.2 ± 12.0 | 62.6 ± 12.3 | 0.004 |
| Sex, male | 542 (86.8) | 1,992 (86.6) | 0.006 |
| Comorbidities |  |  |  |
| Hypertension | 280 (44.8) | 1,038 (45.1) | 0.006 |
| Diabetes mellitus | 178 (28.5) | 667 (29) | 0.010 |
| Cardiovascular disease | 146 (23.4) | 520 (22.6) | 0.017 |
| Ischemic heart disease | 121 (19.4) | 448 (19.4) | 0.001 |
| Valvular heart disease | 4 (0.5) | 7 (0.3) | 0.037 |
| Arrhythmia | 21 (3.2) | 74 (3.2) | 0.003 |
| Heart failure | 14 (2.2) | 64 (2.8) | 0.039 |
| Cerebrovascular disease | 48 (7.7) | 179 (7.8) | 0.004 |
| Chronic kidney disease | 37 (6.0) | 129 (5.6) | 0.017 |
| Chronic lung disease | 19 (3.1) | 71 (3.1) | 0.002 |
| Characteristics of cardiac arrest |  |  |  |
| Place of cardiac arrest |  |  | 0.006 |
| Public place | 214 (34.2) | 784 (34.1) |  |
| Non-public place | 278 (44.5) | 1,031 (44.8) |  |
| Unknown | 132 (21.2) | 486 (21.1) |  |
| Witness type |  |  | 0.015 |
| Healthcare professional | 106 (16.9) | 377 (16.4) |  |
| Bystander | 370 (59.4) | 1,373 (59.7) |  |
| Unknown | 148 (23.7) | 552 (24.0) |  |
| Layperson witnessed | 490 (78.5) | 1,807 (78.6) | 0.002 |
| Bystander-performed CPR | 322 (51.6) | 1,183 (51.4) | 0.003 |
| Telemetric monitoring before EMS | 427 (68.4) | 1,574 (68.4) | <0.001 |
| Initial EMS rhythm |  |  | 0.011 |
| Shockable rhythm | 459 (73.5) | 1,695 (73.7) |  |
| Nonshockable rhythm | 155 (24.8) | 571 (24.8) |  |
| Unknown | 10 (1.7) | 35 (1.5) |  |
| AED usage | 482 (77.3) | 1,785 (77.6) | 0.008 |
| Arrest to admission timeline |  |  |  |
| Arrest-to-call time, min | 0 (0-2) | 0 (0-2) | 0.011 |
| Call-to-door time, min | 30 (24-38) | 31 (26-38) | 0.006 |
| Arrest-to-door time, min | 30 (23-39) | 31 (25-40) | 0.071 |
| PCI timeline |  |  |  |
| Door-to-PPCI time, min | 99 (73-139) | 107 (74-162) | 0.064 |
| Procedure time, min | 23 (14-41) | 23 (13-37) | 0.069 |
| Characteristics on ER admission |  |  |  |
| Initial ECG rhythm |  |  | 0.027 |
| Post ROSC rhythm | 318 (51.0) | 1143 (49.7) |  |
| Shockable rhythm | 71 (11.3) | 263 (11.4) |  |
| Non-shockable rhythm | 178 (28.6) | 674 (29.3) |  |
| No record | 57 (9.2) | 220 (9.6) |  |
| Defibrillation at ER | 208 (33.3) | 762 (33.1) | 0.004 |
| Total CPR time at ER, min | 4 (0-19) | 4 (0-19) | 0.012 |
| ROSC before ER admission | 289 (46.3) | 1,041 (45.2) | 0.021 |
| Therapeutic interventions |  |  |  |
| ECMO | 133 (21.3) | 482 (20.9) | 0.010 |

Values are mean ± SD or n (%).

AED, automated external defibrillator; CPR, cardiopulmonary resuscitation; ECG, electrocardiogram; ECMO, extracorporeal membrane oxygenation; EMS, emergency medical service; ER, emergency room; PCI, percutaneous coronary intervention; ROSC, return of spontaneous circulation; SMD, standardized mean difference.

**Table S3. Comparison of clinical outcome according to the target temperature**

|  | Target temperature, 33℃  (N=523) | Target temperature, 36℃  (N=101) | Unadjusted | | ^*^Multivariable-adjusted | | IPTW-adjusted | |  | |
| --- | --- | --- | --- | --- | --- | --- | --- | --- | --- | --- |
|  |  |  | OR (95% CI) | *P* value | OR (95% CI) | *P* value | OR (95% CI) | *P* value |  |  |
| Mortality outcomes |  |  |  |  |  |  |  |  |  |  |
| Death within discharge | 183 (35.0) | 36 (35.6) | 0.97 (0.63-1.53) | 0.900 | 1.29 (0.75-2.27) | 0.367 | 1.06 (0.82-1.38) | 0.641 |  |  |
| Neurologic outcomes |  |  |  |  |  |  |  |  |  |  |
| Poor outcome (CPC 3, 4, 5) | 301 (57.6) | 55 (54.5) | 1.13 (0.74-1.74) | 0.565 | 1.54 (0.88-2.72) | 0.316 | 1.31 (1.04-1.62) | 0.148 |  |  |

^*^Adjusted variable: age, sex, location at cardiac arrest, bystander-witnessed, bystander-performed CPR, telemetric advice to first aid before EMS, initial EMS rhythm, defibrillation before ER admission, ROSC before ER admission, initial ECG rhythm, defibrillation, total CPR time, defibrillation at ER, ECMO.

Values are n (%, cumulative incidence) unless otherwise indicated. CI, confidence interval- CPC, cerebral performance category- CtD, call-to-door, HR, hazard ratio, OR, odds ratio.

CI, confidence interval; CPC, cerebral performance category; CPR, cardiopulmonary resuscitation; ECG, electrocardiogram; ECMO, extracorporeal membrane oxygenation; EMS, emergency medical service; ER, emergency room; **IPTW,** Inverse probability of treatment weighting; OR, odds ratio; ROSC, return of spontaneous circulation.

**Table S4. Comparison of clinical outcome according to the duration of therapeutic hypothermia**

|  | 24 hours  (N=374) | 48 hours  (N=125) | Unadjusted | | ^*^Multivariable-adjusted | | IPTW-adjusted | |  | |
| --- | --- | --- | --- | --- | --- | --- | --- | --- | --- | --- |
|  |  |  | OR (95% CI) | *P* value | OR (95% CI) | *P* value | OR (95% CI) | *P* value |  |  |
| Mortality outcomes |  |  |  |  |  |  |  |  |  |  |
| Death within discharge | 103 (27.5) | 36 (28.8) | 1.06 (0.67-1.66) | 0.786 | 1.11 (0.65-1.88) | 0.708 | 1.05 (0.79-1.41) | 0.731 |  |  |
| Neurologic outcomes |  |  |  |  |  |  |  |  |  |  |
| Poor outcome (CPC 3, 4, 5) | 197 (52.7) | 63 (50.4) | 0.91 (0.61-1.37) | 0.660 | 0.92 (0.56-1.53) | 0.761 | 0.84 (0.66-1.08) | 0.177 |  |  |

^*^Adjusted variable: age, sex, location at cardiac arrest, bystander-witnessed, bystander-performed CPR, telemetric advice to first aid before EMS, initial EMS rhythm, defibrillation before ER admission, ROSC before ER admission, initial ECG rhythm, defibrillation, total CPR time, defibrillation at ER, ECMO.

Values are n (%, cumulative incidence) unless otherwise indicated. CI, confidence interval- CPC, cerebral performance category- CtD, call-to-door, HR, hazard ratio, OR, odds ratio.

CI, confidence interval; CPC, cerebral performance category; CPR, cardiopulmonary resuscitation; ECG, electrocardiogram; ECMO, extracorporeal membrane oxygenation; EMS, emergency medical service; ER, emergency room; **IPTW,** Inverse probability of treatment weighting; OR, odds ratio; ROSC, return of spontaneous circulation.

**Table S5. Key randomized controlled trials on hypothermia in patients with OHCA**

|  | Study population | Sample size  (Study type) | Comparison | Center (Country) | Primary and key secondary endpoint |
| --- | --- | --- | --- | --- | --- |
| Bernard et al. 2002^22^ | OHCA with VF | 43 vs. 34  (Random) | Hypothermia (33℃) vs. Normothermia (37℃) | 4  (Australia) | *Survival to hospital discharge: 49% vs. 26% (adjusted OR, 5.25; 95% CI, 1.47 to 18.76) |
| HACA trial 2002^23^ | OHCA with VF | 137 vs. 138  (Random) | Hypothermia (32-34℃) vs. Normothermia | 8  (Europe) | *Favorable neurologic outcome (CPC 1 or 2) within 6 months: 55% vs. 39% (RR, 1.40; 95% CI, 1.08 to 1.81)  Mortality at 6 months: 41% vs. 55% (RR, 0.74; 95% CI, 0.58 to 0.95) |
| TTM trial 2013^24^ | OHCA of presumed cardiac cause | 473 vs. 466  (Random) | Hypothermia (33℃) vs. Normothermia (36℃) | 36 (Europe and Austria) | *Death at end of trial: 50% vs. 48% (HR, 1.06; 95% CI, 0.89 to 1.28)  Poor neurologic function (6-month): 54% vs. 52% (HR, 1.02; 95% CI, 0.88 to 1.16) |
| TTH48 trial 2017^25^ | OHCA | 176 vs. 179 (Random) | Hypothermia (33℃) for 48 hrs vs. 24 hrs | 10 (Europe) | *Favorable neurologic outcome (CPC 1 or 2) within 6 months: 69% vs. 64% (RR, 1.08; 95% CI, 0.93 to 1.25)  Mortality at 6 months: 27% vs. 34% (RR, 0.81; 95% CI, 0.59 to 1.11) |
| HYPERION trial 2019^26^ | OHCA with nonshockable rhythm | 284 vs. 297  (Random) | Hypothermia (33℃) vs. Normothermia (37℃) | 25  (France) | *Favorable neurologic outcome (CPC 1or 2) at 90 days: 10.2% vs. 5.7% (HR, 4.5; 95% CI 0.1 to 8.9)  Survival to hospital discharge: 19.7% vs. 16.8% (HR, 1.19; 95% CI, 0.81 to 1.74) |
| TTM2 trial 2021^27^ | OHCA | 930 vs. 931  (Random) | Hypothermia (33℃) vs. Normothermia (37.5℃) | 65  (Global) | *Death at 6 months: 50% vs. 48% (RR, 1.04; 95% CI, 0.94 to 1.14)  **Poor functional outcome at 6 months: 55% vs. 55% (RR, 1.00; 95% CI 0.92 to 1.09) |

* indicates primary endpoint
** Poor functional outcomes was defined as modified Rankin scale score 4 to 6
AMI, acute myocardial infarction; BOX, Blood Pressure and Oxygenation Targets in Post Resuscitation Care; CA, cardiac arrest; CI, condidence interval; CPC, Cerebral Performance Category; CS, cardiogenic shock; GOS, Glasgow Outcome Scale; HACA, Hypothermia After Cardiac Arrest; HR, hazard ratio; HYPERION, Therapeutic Hypothermia After Cardiac Arrest in Non Shockable Rhythm; OHCA, out-of-hospital cardiac arrest; OR, odds ratio; PCI, percutaneous coronary intervention; RR, relative risk; SHOCK-COOL, Mild Hypothermia in Cardiogenic Shock Complicating Myocardial Infarction; TTH48, Time-differentiated Therapeutic Hypothermia; TTM, Target Temperature Management 33°C versus 36°C after Out-of-Hospital Cardiac Arrest; TTM2, Targeted Hypothermia Versus Targeted Normothermia After Out-of-hospital Cardiac Arrest; VF, ventricular fibrillation

**Figure S1. Propensity score distribution before (A) and after IPTW (B)**

**
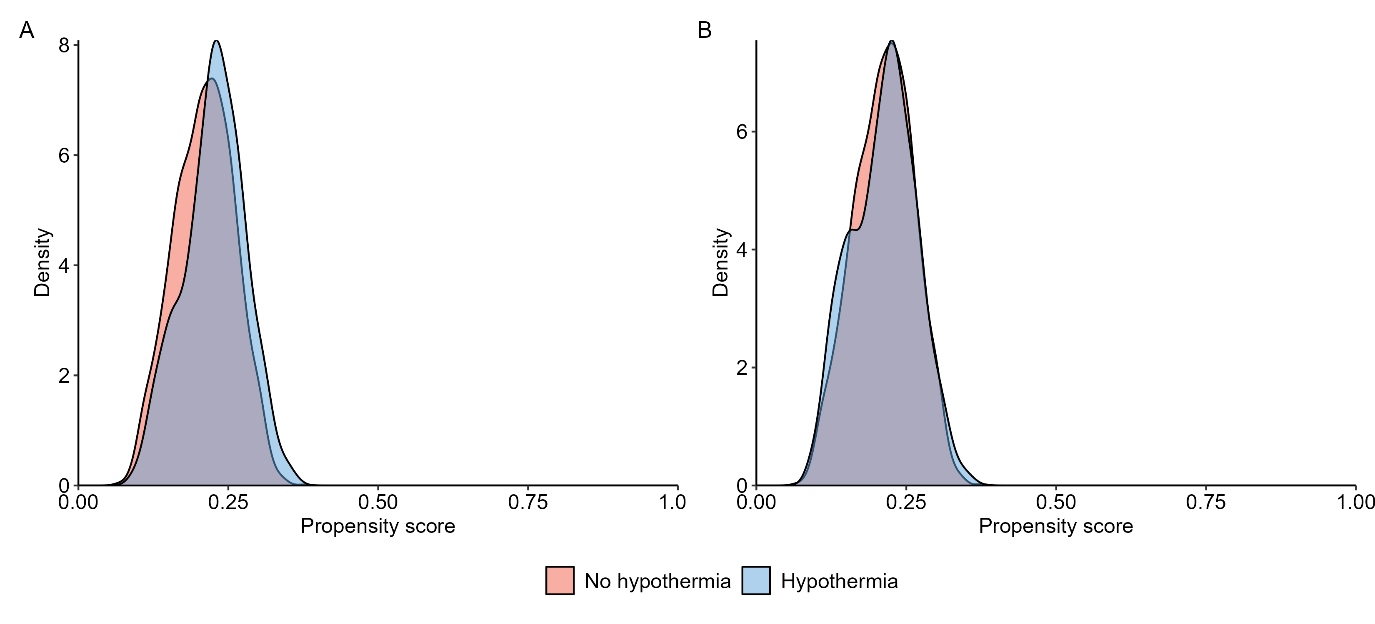
**

**Figure S2. Subgroup analysis for in-hospital mortality and poor neurological outcomes**


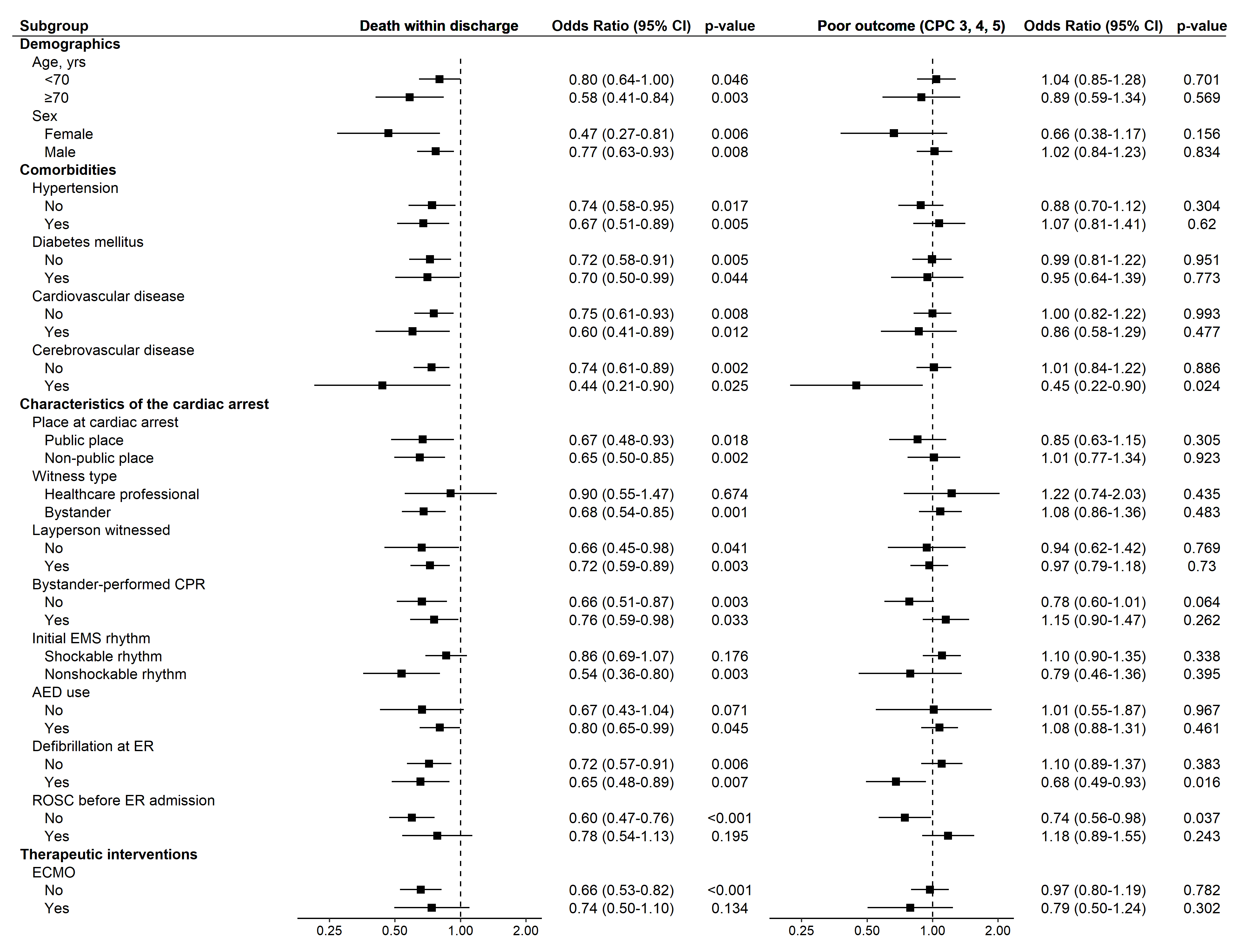


CPC, cerebral performance category; CPR, cardiopulmonary resuscitation; EMS, emergency medical service; AED, automated external defibrillator; ER, emergency room; ROSC, return of spontaneous circulation; ECMO, extracorporeal membrane oxygenation.
